# Organisational perspectives on Public Health delivery in Ireland: Lessons learned from COVID-19

**DOI:** 10.1093/eurpub/ckac129.208

**Published:** 2022-10-25

**Authors:** M Norris, P Carty, C McDowell, M O’Loughlin, F Comaskey, J Jingjing, P Harrington, M Ryan, M O'Neill

**Affiliations:** 1 Health Technology Assessment, Health Information and Quality Authority, Dublin, Ireland; 2 Department of Public Health, Health Service Executive Midwest, Limerick, Ireland; 3 Department of Pharmacology and Therapeutics, Trinity College Dublin, Dublin, Ireland

## Abstract

**Background:**

The COVID-19 pandemic has placed healthcare systems worldwide under unprecedented pressure, with the Irish Public Health system no different. To strengthen delivery of Essential Public Health Functions (EPHFs) and increase future pandemic preparedness, Public Health leaders are now focused on identifying learnings from the pandemic. Within Ireland, given their experience, organisations situated within the Public Health system may be in a unique position to provide valuable information around the delivery of EPHFs, both prior to and in light of the COVID-19 pandemic, and how this can be improved in the future.

**Methods:**

An online survey was distributed by the Department of Health, from 2 March 2022 to 25 March 2022, amongst organisations situated within the Public Health domain in Ireland. The survey consisted of six open-ended questions around the delivery of EPHFs prior to and in light of the pandemic, success stories that could provide scalable solutions to EPHF delivery and current health system barriers, key areas in the public health system that require strengthening, and barriers to achieving these actions. Thematic analysis to identify key themes was conducted on survey responses.

**Results:**

Twenty-eight organisational responses were received. Themes around the workforce were apparent throughout, with staff training, staff diversity and staff morale, identified as areas for strengthening EPHF delivery. Themes around ICT, data collection and research were frequently identified with a lack of adequate ICT identified as a key lesson from the pandemic, while the Public Health ICT strategy was identified as key to strengthening future EPHF delivery.

**Conclusions:**

In general, themes around the workforce; leadership, management and governance and ICT, data collection and research were reoccurring across organisational responses and therefore may be key areas for consideration when strengthening delivery of the EPHFs in Ireland.

**Speakers/Panelist:**

**Louise Hendricks**

Department of Health, Ireland

**Sohel Saikat**

WHO, Geneva, Switzerland

